# Between Protection and Destruction: The Legal Tension Between Military Necessity and Cultural Heritage Protection in International Humanitarian Law

**DOI:** 10.12688/f1000research.170650.2

**Published:** 2025-10-17

**Authors:** Jinane El Baroudy, Mohamad Albakjaji, Maya Khater

**Affiliations:** 1 College of Law, Prince Sultan University, Riyadh, Riyadh Province, 11461, Saudi Arabia; 2 College of Law, Prince Sultan University, Riyadh, Riyadh Province, 11461, Saudi Arabia; 3 College of Law, Abu Dhabi University, Abu Dhabi, Abu Dhabi, 59911, United Arab Emirates

**Keywords:** Military necessity, Cultural heritage, Proportionality, Destruction, Protection of cultural property, International Humanitarian Law, ICTY, ICC.

## Abstract

**Background:**

The relationship between military necessity and the protection of cultural heritage under international humanitarian law has been and continues to be governed by a constant tension that has shaped both doctrine and practice. While the principle of military necessity, rooted in and derived from the 1863 Lieber Code and codified in later instruments such as the 1907 Hague Conventions, remains one of the most fundamental principles permitting the adoption of measures necessary to achieve legitimate military objectives, it has also been used to justify the destruction of cultural property. Conversely, cultural heritage, deserving special protection under instruments such as the 1954 Hague Convention and its Protocols, represents the collective memory and identity of societies.

**Methods:**

To evaluate the role of military necessity in the prosecution of crimes against cultural heritage, this paper uses a critical doctrinal and analytical approach, looking at both treaty law and international jurisprudence. It asks whether military necessity is a valid practical safeguard or a legal pretext for destruction.

**Results:**

The paper contends that the incorporation of “imperative military necessity” has engendered critical legal loopholes resulting from warring parties’ exploitation of the prioritization of military interests over the preservation of cultural heritage, rendering cultural heritage vulnerable in armed conflicts. International tribunals, such as the International Criminal Tribunal for the Former Yugoslavia (ICTY) and the International Criminal Court (ICC), have endeavoured to restrict these justifications by criminalizing deliberate attacks and applying standards of necessity and proportionality.

**Conclusions:**

The paper concludes that, despite treaties and jurisprudence limiting the scope of military necessity, the ongoing recognition of the necessity exception reveals structural weaknesses in international humanitarian law, thereby rendering cultural heritage vulnerable in armed conflict. Accordingly, it advocates for the strict limitation of the ‘imperative military necessity’ exception to provide better protection for cultural heritage during armed conflict.

## Introduction

In international humanitarian law (IHL), the relationship between military necessity and the protection of cultural heritage is marked by a persistent and, at times, irreconcilable tension. This dynamic is not incidental but is deeply embedded in the fabric of IHL’s treaty framework. The notion of military necessity has deep historical roots, first expressed in the United States’ Lieber Code of 1863—where it was described as measures “indispensable for securing the ends of war, and lawful according to the modern law and usages of war”—and later codified in the 1907 Hague Conventions ( UNESCO, 1995). Military necessity, as a fundamental principle, permits measures deemed essential for achieving legitimate military objectives and is characterized as “a basic principle of the law of war, so basic, that without it, there could be no law of war whatsoever.” ( Forrest, 2007, p. 183). Yet, in practice, military necessity has repeatedly been invoked to justify actions detrimental to civilian belongings, including cultural property ( Khater, 2022a, 2022b). In stark contrast, cultural heritage represents the collective memory and identity of societies, embodied in monuments, artifacts, and sites of historical, artistic, and religious significance. When these two principles come into conflict, legal frameworks have too often tilted toward military expediency at the expense of preservation, leaving the cultural patrimony of humanity exposed to destruction under the guise of necessity.

The 1954 Hague Convention for the Protection of Cultural Property in the Event of Armed Conflict stands as the cornerstone of international efforts to safeguard cultural heritage. It codifies concrete protective obligations and introduces the Blue Shield emblem as a means of ensuring the recognition and respect of such property during hostilities. Yet, this cornerstone treaty permits derogation in cases of “imperative military necessity,” a loophole that belligerents have exploited to justify attacks on sites of profound historical and cultural significance. Subsequent instruments, such as the 1977 Additional Protocols and the 1999 Second Protocol, have attempted to clarify these protections by precisely defining the circumstances under which military necessity may override preservation, particularly under Article 6, which restates the principles concerning military objectives outlined in Additional Protocol I ( Protocol Additional I to the Geneva Conventions, 1977, art. 52(2)). Still, the language of these treaties remains narrowly defined and strictly conditional ( Second Protocol to the Hague Convention of 1954, 1999, art. 6(a)). Which, although intended to limit discretionary interpretations, effectively permits states and military commanders to prioritize military objectives over the protection of cultural heritage ( Johannot-Gradis, 2015).

The practical consequences of this tension are profound. International courts and tribunals—from the ICTY to the ICC—have been compelled to confront the challenge of regulating a principle that is both conceptually ambiguous and operationally far-reaching. The Rome Statute explicitly criminalizes deliberate attacks on cultural property, nonetheless, tribunals have repeatedly encountered claims invoking military necessity as a justificatory defense. Cases such as Blaškić, Strugar, and Hadžihasanović demonstrate that cultural property might only lose protection when it is contemporaneously used for military purposes, and even in such instances, proportionality must be rigorously assessed. The ICC’s ruling of Timbuktu’s mausoleums in Prosecutor v. Al Mahdi underscores the principle that heritage possesses basic value independent of military considerations. However, the persistent presence of the military necessity exception continues to weaken the enforcement of these protective norms.

The aim of this paper is to critically examine the role of military necessity in prosecuting crimes against cultural heritage, questioning whether it operates as a legitimate operational safeguard or merely a legal pretext for the destruction of irreplaceable cultural property. The coexistence of military necessity and cultural heritage raises critical questions: to what extent can military necessity justify attacks on cultural property, and how does it influence prosecution? Drawing on treaty provisions, jurisprudence, and scholarly analysis, this paper assesses how the international law reconciles operational imperatives with heritage protection, highlighting the structural vulnerabilities in IHL that allow broadly defined exceptions to be invoked in ways that may legitimize war crimes.

The paper seeks to (1) analyze military necessity as an underlying doctrinal issue, investigating its historical misuse and its disruptive potential in weakening cultural property protections. And (2) evaluate the limited impact of military necessity in practice, exploring how treaties and judicial decisions have limited their application while reinforcing the obligation to preserve heritage. Collectively, these sections provide a dual perspective, highlighting both the disruptive potential and the circumscribed role of military necessity within the framework of international humanitarian law.

## Methods

### Study design

This paper adopts a descriptive, critical, and analytical legal design. It assesses the relationship between military necessity and the protection of cultural heritage from the perspective of applicable international humanitarian law.

### Data collection

The paper primarily relies on major international treaties, such as the 1863 Lieber Code, the 1907 and 1954 Hague Conventions, the 1999 Second Protocol, the 1977 Additional Protocols, and the Rome Statute of the International Criminal Court (Rome Statute). It also examines the case law of international tribunals, particularly the International Criminal Tribunal for the Former Yugoslavia (ICTY) and the International Criminal Court (ICC), to further assess the practical application of these sources.

### Data analysis

The paper uses a comparative and critical analytical method to examine these sources. Each treaty provision is considered with concomitant judicial meanings to ascertain to what extent military necessity is a vehicle for cultural destruction or a protective device. More specifically, the study examines the proportionality principle, the definition of military objectives, and the jurisprudential boundaries established for the doctrine of military necessity. In this manner, the study identifies structural flaws and legal gaps within international humanitarian law that make cultural heritage susceptible during armed conflict.

## Results

The paper highlights several important findings regarding the relationship between military necessity and cultural property protections under the international humanitarian law. To begin with, the military necessity exception in the 1954 Hague Convention and its protocols creates a legal gap. Intended to be precisely defined, it empowers states and commanders to justify attacks on cultural property and the military advantages that contributed to both the identification of the objective and the stated purpose.

In this regard, the 1999 Second Protocol attempted to identify limits for military necessity under the military objectives and feasibility alternatives provisions. However, its vague and ambiguous wording renders cultural property susceptible to exploitation in times of armed conflict, thereby undermining the protective framework it intended to establish.

In parallel, international tribunals, such as the ICTY and the ICC have sought to restrict the military necessity defense. Cases such as Strugar and Al Mahdi confirmed that cultural property is protected against assault unless it is actively employed for military objectives, in which case, it must undergo strict proportionality assessments. Further, despite the tribunal’s attempts, the inconsistent application across cases (i.e. Prlić) further illustrates the tenuousness of these protections.

Moreover, while proportionality is meant to function as a protective measure, it ultimately operates in a manner that prioritizes military objectives over culture preservation. This imbalance has legitimized the destruction of cultural heritage under the guise of legitimate military necessity.

To conclude, the persistence of the necessity exception points to systemic weaknesses in international humanitarian law. Cultural heritage remains subject to operational convenience, resulting in a lack of legal accountability and effective deterrence.

## Discussion

### Military Necessity as a Challenge to Cultural Heritage Protection

The 1954 Hague Convention, widely regarded as the cornerstone of cultural property protection, contains a military necessity clause—Article 4(2)—that permits derogation “in cases where military necessity imperatively requires such a waiver.” While this provision underscores a persistent vulnerability in the regime, it theoretically presumes a high evidentiary threshold, as “imperative necessity” requires compelling proof before a waiver can be legitimately invoked ( von Schorlemer, 2004). Nonetheless, it provides a potential basis for State Parties to justify actions whenever military objectives can be plausibly framed ( UNESCO, 1995). Central to the Convention, Article 4 mandates State Parties to refrain from using cultural property—or its immediate surroundings—in ways likely to expose it to destruction or damage during armed conflict. More broadly, parties must abstain from any acts of hostility against such property, implement protective measures, and avoid reprisals targeting cultural heritage. These obligations apply not only to States safeguarding property within their own territories but also to those engaged in conflicts where cultural heritage is at risk ( Forrest, 2007).

**Legal Framework of Military Necessity from the 1954 Hague Convention to the 1999 Second Protocol**

Against this backdrop, the second paragraph of Article 4 introduces a crucial exception ( Forrest, 2007). Although it constitutes the sole deviation from otherwise clear rules of protection, it also poses the central challenge of reconciling the imperative to safeguard cultural property with the perceived necessity of conducting military operations during armed conflict ( Ryška, 2021). Military necessity is neither precisely defined nor objectively delimited, making it vulnerable to misuse as a justification for attacks based on subjective assessments of military advantage ( Pavoni, 2020; El Baroudy & Albakjaji, 2024). Critically, any use of force that is not genuinely necessary remains unlawful and also military necessity does not provide carte blanche to violate the laws of armed conflict. The doctrine of *kriegsraison* has been explicitly rejected—military necessity cannot serve as a basis for unrestricted warfare, even under extreme circumstances. By definition, the principle of military necessity is inseparable from the principles of humanity and proportionality ( Ryška, 2021). Accordingly, whether an act is mandated by military necessity requires a proportionality assessment ( Gardam, 2004), any use of cultural property that exposes it to destruction or damage, or any hostile action affecting it, must be proportionate to the concrete and direct military advantage anticipated. Nevertheless, the principle also opens the door to potential justification for the destruction of cultural property, elevating such acts to a sphere of possible legitimacy ( Forrest, 2007). Given its flexible and “open-textured” nature, the “military necessity” remains susceptible to misuse as a justification for offenses against cultural property based on subjective assessments of military convenience ( Pavoni, 2020).

Building on these safeguards, the 1954 Hague Convention introduced the concept of *special protection* to enhance the protection of cultural property of exceptional importance ( Convention for the Protection of Cultural Property in the Event of Armed Conflict, 1954). To ensure recognition, the emblem for specially protected property must be displayed during armed conflict, although its placement before hostilities is optional ( Convention for the Protection of Cultural Property in the Event of Armed Conflict, 1954). More precisely, this Status applies to a limited number of refuges for movable cultural property and centers containing monuments or other immovable cultural property of considerable importance, provided they are not used for military purposes, are located at an “adequate distance” from large industrial centers or significant military objectives, and are registered in the International Register of Cultural Property ( Convention for the Protection of Cultural Property in the Event of Armed Conflict, 1954). Despite these provisions, it should be emphasized that the term ‘adequate distance’ is undefined and context-dependent, with scholars such as Toman and O’Keefe advocating a case-by-case approach, highlighting how its vagueness has discouraged States from seeking special protection ( Ryška, 2021). Further clarifying the limits of protection, it is stipulated in Article 11(2) that immunity may be withdrawn only in exceptional cases of unavoidable military necessity ( Convention for the Protection of Cultural Property in the Event of Armed Conflict, 1954), such as when the property lies in the immediate vicinity of military objectives and its protection would seriously hamper operations, or if the defending party breaches its obligations ( Forrest, 2007). Decisions must be made by a division-level commander or higher; reasonable advance notice should be provided when possible, and the withdrawal must last only as long as necessary. It is argued that this regime is overly narrow, particularly due to the distance requirement and limited flexibility regarding which cultural property can be granted special protection ( Von Schorlemer, 2004).

**From Principle to Pretext: The Misapplication of Military Necessity**

Reflecting this concern, the attempt to reconcile military imperatives with the protection of cultural property is further reflected in the elaboration of these principles in the 1999 Second Protocol to the 1954 Hague Convention ( Albakjaji, 2022). Developed in response to the destruction of cultural property in Somalia, Iraq, Kuwait, and the former Yugoslavia, the Protocol provides important guidance on the application of the military necessity clause, clarifying and limiting the circumstances under which derogations may be justified ( Forrest, 2007). Consequently, Military necessity may justify an attack on cultural property only if the property has, through its function, transformed into a military objective and if no feasible alternative exists to achieve a comparable military advantage ( De Vriese, 2018). Article 52(2) of Additional Protocol I and the Second Protocol to the Hague Convention raise two key issues regarding the application of military necessity to cultural property. First, the Second Protocol introduces the concept of cultural property becoming a military objective *“by its function,”* a criterion absent from Additional Protocol I, which considers the nature, location, purpose, or use of the property. This new condition is somewhat vague and complicates its application alongside traditional criteria ( Hladík, 2005). It may be added that experts have observed that military necessity could *“virtually never be invoked to justify an attack on cultural property, since there are almost always feasible alternatives to circumvent the property.”* However, circumvention is required only to the extent that an alternative exists to achieve a similar military advantage ( Second Protocol to the Hague Convention of 1954, 1999, 6(a)(ii)), effectively limiting the number of alternatives a commander must consider before targeting cultural property ( Forrest, 2007). Attention is also drawn to the fact that the Second Protocol thus introduces significant changes, addressing gaps left unresolved in the main Convention ( Second Protocol to the Hague Convention of 1954, 1999, 6(b); Toman, 1996; Gerstenblith, 2015). In this context, imperative military necessity only applies when cultural property is employed as a military objective and *“no feasible alternative exists to achieve a similar military advantage.”* ( Forrest, 2007, p. 211; Second Protocol to the Hague Convention of 1954, 1999, art. 6(a)). Consequently, the use of cultural property in ways that might expose it to damage or destruction is permissible only *“when and for as long as no alternative method exists to obtain a comparable military advantage.”* ( Second Protocol to the Hague Convention of 1954, 1999, 6(b)). Under present-day definitions, military objectives comprise objects that may lawfully be targeted in accordance with international humanitarian law. Generally, a military objective is defined as an object which, by its *nature, location, purpose, or* use, makes an effective contribution to military action and whose destruction, capture, or neutralization, the circumstances ruling at the time, offers a definite military advantage ( Second Protocol to the Hague Convention of 1954, 1999, 6(b)). In this regard, this definition encompasses two key elements, namely the inherent characteristics of the object—its nature, location, purpose, or use—and the anticipated military advantage resulting from its attack ( Fenrick, 2009). As one might expect, an attack on an ammunition dump is universally recognized as a lawful military objective, whereas a school occupied only by children and teachers is decidedly off-limits ( Fenrick, 2009). Uncertainty persists as to how the definition applies to dual-use objects serving both civilian and military functions, and how flexibly—or even opportunistically—its application may shift in response to the changing dynamics of armed conflict ( Henckaerts, 1999). It becomes clear that it is long overdue to prevent the exploitation of military necessity as a pretext for attacks. When multiple military objectives exist and one is a cultural object, that cultural property must not be targeted. Only legitimate military objectives may be attacked; non-military objects, including cultural property, are strictly protected, underscoring the essential duty to safeguard humanity’s heritage even in times of war ( von Schorlemer, 2004). In line with this reasoning, any improvement to the 1954 Hague Convention should reflect the modern principle that cultural property is presumptively civilian and may be attacked only if it becomes a military objective. Yet this formulation is hardly reassuring, since it leaves open the very loophole that has historically legitimized destruction under the banner of necessity, raising the question of whether the Convention strengthens protection or merely redefines the terms of its erosion. Although the adoption of the 1999 Second Protocol truly closed these gaps, it remains an open and contested question. While it introduced stricter conditions for invoking military necessity and expanded individual criminal responsibility ( Henckaerts, 1999), its effectiveness in practice might be far from settled. On the one hand, the Protocol seeks to clarify when cultural property may be legitimately targeted, yet on the other, it could illustrates the uneasy balance that humanitarian law continues to strike between military imperatives and humanitarian considerations.

Significantly, the incorporation of ‘military advantage’ in the definition of military objectives under Article 52(2) of Additional Protocol I and the Second Protocol to the Hague Convention adds further tension to the protective framework. Consequently, although the concept of military advantage is central to determining legitimate targets, it offers limited constraint on invoking exceptions. Many cultural properties may have historical military associations, yet their nature, purpose, or location alone does not render them lawful targets unless they are actively used for military purposes ( Hladík, 2005). It should be noted, however, that the waiver permitting the use of cultural property for military purposes may only be invoked when no feasible alternative exists to achieve a comparable military advantage. Craig Forrest notes; “the concept of a military advantage … *appears to offer no limitation on the grounds upon which the adequate distance exception can be raised,” (* *Chechi & Romani, 2023* *,15) highlighting* the ongoing tension between military necessity and the protection of cultural heritage.

Similarly, an opposing belligerent state is required to take a number of precautions to avoid targeting cultural property. ( Second Protocol to the Hague Convention of 1954, 1999, 7). With due regard to military advantage, a State Party must “do everything feasible to verify that the objectives to be attacked are not cultural property protected under … the Convention” and must “take all feasible precautions in the choice of means and methods of attack with a view to avoiding or minimize incidental damage.” ( Second Protocol to the Hague Convention of 1954, 1999, art. 7 (a)-(b)). It should be ensured that any attack likely to cause incidental damage is not carried out if such damage would be ‘excessive in relation to the concrete and direct military advantage anticipated’ ( Forrest, 2007). Without going into a detailed explanation, it is important to note that necessity and advantage are not synonymous. Accordingly, the latitude afforded by these provisions is framed in terms of achieving a military advantage, effectively prioritizing acts or omissions that favor operational convenience over the protection of cultural property. As a result, in practice, the concept of military necessity is reduced to mere military advantage, which affects the application of proportionality by weighing humanitarian considerations against military gain rather than genuine necessity. This is most evident in the provision on the military necessity exception, wherein international law not only permits but effectively legitimizes the destruction of cultural heritage under the guise of legality—functioning less as a safeguard than as a pretext for cultural destruction ( Second Protocol to the Hague Convention of 1954, 1999).

**Military Necessity, Proportionality, Legal Loopholes in Cultural Heritage Protection**

The Second Protocol further codifies several key obligations in Article 7, foremost among them the principle of proportionality. This principle requires a party to refrain from conducting any attack expected to cause incidental damage that would be excessive in relation to the concrete and direct military advantage anticipated. Furthermore, State Parties are legally obligated to take all feasible measures to limit incidental damage and to adopt precautions to prevent the accidental targeting of cultural property ( Second Protocol to the Hague Convention of 1954, 1999). Yet in practice, these obligations are frequently ignored, making cultural heritage vulnerable under the pretext of military operations ( Alsamara, Ghazi, & Mallaoui, 2022). It is important to clarify the meaning of ‘proportionality,’ which in international humanitarian law is defined in strikingly limited wording: it does not concern the imbalance of military power between parties, but merely whether incidental harm is deemed excessive in relation to the anticipated military advantage. This interpretation is legally irrelevant for targeting decisions and risks obscuring the real issue. In targeting law, proportionality requires weighing expected civilian or cultural harm against the concrete and direct military advantage anticipated. Framed in this way, the principle is structurally skewed: it entrenches military advantage as the decisive factor while relegating cultural protection to a negotiable cost, thereby legitimizing destruction rather than preventing it. Similarly, despite the clear standard set by Article 51 of Additional Protocol I—prohibiting indiscriminate attacks and classifying certain forms under Article 51(5)(b) as inherently indiscriminate—these protections are often disregarded in practice, effectively emptying the provision of its force. Consequently, the safeguarding of civilians and cultural heritage becomes contingent upon operational convenience, allowing destruction to be legally tolerated rather than genuinely prevented ( Fenrick, 2009). It must be recognized that the core challenge lies not in the principle of proportionality itself, but in its practical application, which demands balancing legitimate destructive effects against undesirable collateral consequences. This assessment is inherently difficult and often subjective, as it requires comparing incommensurable values—such as the loss of innocent human lives versus the capture or destruction of a military objective—opening the door to misuse under the guise of legality ( Fenrick, 2009; Watkin, 2005). It must be acknowledged that the precise relationship between military necessity and proportionality remains somewhat ambiguous. While the two concepts are widely recognized as related, there is no agreement on their practical interaction. Some scholars treat proportionality as part of military necessity, while others consider military necessity an element of proportionality, highlighting not only the conceptual difficulties but also the risk that this ambiguity can be exploited to justify attacks on cultural property ( Hayashi, 2010). Furthermore, the issue of legitimate targeting of cultural property reveals the conflict between military objectives and humanitarian obligations. States must weigh the anticipated military advantage against potential excessive damage, a task complicated by incomplete knowledge and rapid operational decision-making ( Johannot-Gradis, 2015). A striking example from the First Gulf War shows that during the 1991 campaign in Iraq, an Iraqi aircraft was strategically positioned next to the 2,000-year-old Temple of Ur to protect it from attack. The aircraft was a legitimate target, although its destruction offered minimal military advantage, while the potential harm to the temple would have been clearly disproportionate. This case highlights both the practical difficulty of applying proportionality and the tendency for military expediency to supersede the protection of cultural heritage ( Johannot-Gradis, 2015). The principle of proportionality under Article 7 requires weighing incidental harm against anticipated military advantage, yet its narrow definition frequently skews this balance in favor of military gain. Historical and contemporary examples illustrate this tension. During the First Gulf War, an Iraqi aircraft was strategically positioned near the 2,000-year-old Temple of Ur—a legitimate target whose destruction offered minimal military advantage—yet the potential harm to the temple would have been clearly disproportionate, demonstrating the difficulty of applying proportionality in practice ( Johannot-Gradis, 2015). Similarly, in 2001, the Taliban destroyed the Bamiyan Buddhas in Afghanistan, claiming military and political justification despite no tangible military benefit, highlighting the risk that military or political motives can masquerade as necessity ( Techera, 2007). Going further back, the Allied bombing of Dresden during World War II sparked debates over military necessity versus the disproportionate destruction of civilian life and cultural property, showing that the tension between operational objectives and humanitarian obligations is not only contemporary but historically entrenched ( Fenrick, 2009; Watkin, 2005).

Military necessity is intended to set a high threshold, although its application is still limited. Article 6 of the Second Protocol restrict the use of this exception to senior officers commanding battalion-sized forces or higher, requires reasonable advance warning where feasible ( Techera, 2007; Afriansyah, 2013), and mandates a two-part reasonableness test ( Forrest, 2007). This test includes an objective assessment of intelligence gathered prior to the attack and a subjective evaluation of the commander’s actions based on available information ( Second Protocol to the Hague Convention of 1954, 1999) While involving senior officers aims to ensure more objective decisions, it may provide only limited protection, as choices can still favor overall military advantage rather than immediate necessity. Moreover, the absence of guidance for junior officers further weakens safeguards for cultural property, leaving it exposed to operational convenience Critically, the incorporation of military necessity exceptions undermines the protection of cultural heritage in favor of operational priorities ( Techera, 2007; Afriansyah, 2013). This measure elevates operational considerations above humanitarian protections. It has been widely observed that easing the invocation of military necessity might directly weaken the protection of cultural property ( Khater, 2025). As Toman observes, some scholars argue that removing such exceptions would reinforce humanitarian law by ensuring that the burden of proof remains on parties seeking to justify the destruction. Yet, while the 1999 Second Protocol strengthens cultural property protections in many ways, its military necessity clause might paradoxically allow attacks under specific conditions, effectively placing military objectives above humanitarian safeguards. By contrast, in contexts where international humanitarian law makes no reference to military necessity, protection is absolute, permitting no derogation ( Carrijo & Squeff, 2024).

### The Limited Impact of Military Necessity on Protecting Cultural Heritage in Armed Conflict

International law establishes a fundamental rule that cultural property must never be deliberately targeted during military operations in both international and non-international armed conflicts. This prohibition is firmly rooted in customary international law and binds even States that have not ratified the relevant treaties. The protection is further reinforced by the Rome Statute of the International Criminal Court (1998), which under Article 8 classifies as war crimes any intentional attacks against buildings dedicated to religion, education, art, science, or charitable purposes, as well as historic monuments, provided these sites are not misused for military purposes ( Pocar, 2024). However, this seemingly absolute protection is undermined by the doctrine of “military necessity,” which acts as a corrosive exception. The doctrine is formally limited to measures indispensable for achieving legitimate war objectives, in accordance with the laws and customs of war ( ICTY, *Prosecutor v. Kordić & Čerkez*, 2001). In practice, it legitimizes operations that unavoidably cause civilian harm, including injury and death, treating such consequences as tolerable byproducts of combat ( Khater, 2023). Most concerningly, it allows the deliberate destruction of cultural property whenever “imperative” justification is claimed, effectively transforming a protective regime into one that allows cultural heritage to be sacrificed on the altar of military convenience ( ICTY, *Prosecutor v. Galić*, 2003a).

**Shaping the Doctrine of Military Necessity in International Law and ICTY Practice**

First, the ICTY progressively sharpened its stance on military necessity in relation to attacks on cultural heritage and civilian property. However, its jurisprudence exposes the ongoing conflict between law and convenience. In landmark cases such as *Blaškić* and *Kordić* and *Čerkez* in 2001, the ICTY claimed that criminalizing such attacks requires two conditions: the site must not be used for military purposes at the time and must not be in the immediate vicinity of military objectives ( De Vriese, 2018). This “military purposes” standard, derived from the Hague Regulations, theoretically offered stronger protection than the Rome Statute’s broader “military objective” standard, which risks sanctioning attacks even where defenders have not used the site militarily. However, this strict criterion, particularly problematic for cultural sites in urban areas, was gradually diluted, leaving heritage exposed to operational convenience ( Brammertz *et al.*, 2016). In *Galić case*, the Tribunal asserted that an object cannot lawfully be attacked if, based on available information, it is unreasonable to believe that it effectively contributes to military action ( ICTY, *Prosecutor v. Galić*, 2003a). Similarly, in cases such as *Naletilić* and *Martinović* in 2003, the Chamber stressed that mere proximity to military targets does not justify the destruction of cultural property, yet protection is still lost once the property is pressed into military use ( ICTY, *Prosecutor v. Naletilić & Martinović*, 2003b). By contrast, *Strugar* adopted a more heritage-sensitive approach, holding that sites near, but not themselves constituting, military objectives cannot be targeted ( ICTY, *Prosecutor v. Naletilić & Martinović*, 2003b). This stance was reaffirmed in *Martić* case in 2007, where the Tribunal declared that the decisive factor is the site’s actual military use at the time. Even in that case, the attack on two churches was not considered criminal as they were exploited for military purposes during hostilities ( ICTY, *Prosecutor v. Strugar*, 2003c). The Chamber nevertheless emphasized that the military-use exception is applicable even when the sites are an integral part of a community’s cultural or spiritual identity, exposing the troubling reality that international law, under the guise of legitimacy, can be exploited to justify the destruction of cultural heritage in the name of military expediency ( Drazewska, 2022; ICTY, *Prosecutor v. Martić*, 2007). The ICTY’s jurisprudence starkly revealed the fragility of legal protections for cultural heritage. Trial Chambers at times suggested that attacks on civilians or civilian property might be justified under military necessity. Nonetheless, these flawed findings were overturned on appeal, with the Appeals Chamber reaffirming the absolute prohibition under Article 52(2) of Additional Protocol I against targeting civilians and civilian objects. Nevertheless, the very reliance on the notion of “military necessity,” coupled with the required nexus to armed conflict, created a high threshold for effective protection ( Abtahi, 2015). In *Hadžihasanović* case, the Tribunal acknowledged this tension while distinguishing its approach from Additional Protocol I, which does not permit any waiver based on military necessity ( De Vriese, 2018; ICTY, *Prosecutor v. Hadžihasanović & Kubura*, 2006). Article 2(d) nominally allows the destruction of civilian property when military necessity dictates, effectively subordinating the protection of cultural sites to operational expediency ( O’Keefe, 2010). Its scope is inherently limited, hinging on the vague and malleable concept of military necessity. The critics have rightly argued that the notion is imprecise, inconsistently applied, and outdated, systematically placing cultural preservation below military aims and eroding the Hague Convention’s vision of cultural property as humanity’s shared inheritance ( De Vriese, 2018). In *Prlić et al.,* the ICTY confronted these limitations when assessing the destruction of the Old Bridge of Mostar ( Drazewska, 2022). Although the bridge was employed for military purposes, the Trial Chamber found its destruction unlawful, holding that the resulting harm to civilians and cultural heritage was disproportionate to any military advantage gained ( ICTY, *Prosecutor v. Prlić et al.*, 2013). The Chamber thus applied a proportionality test, confirming that cultural objects pressed into military use retain protection ( Drazewska, 2022; ICTY, *Prosecutor v. Prlić et al.*, 2013) unless an attack is truly proportionate—a condition the bridge’s destruction did not meet ( ICTY, *Prosecutor v. Prlić et al.*, 2013). Yet the 2017 Appeals Chamber overturned the convictions for wanton destruction without engaging with proportionality or military necessity ( Cotter, 2018), asserting merely that destroying a military target could not be “unnecessary” since it offered a “definite military advantage.” ( ICTY, *Prosecutor v. Prlić et al.*, 2017a, para. 411) This reasoning dangerously conflated military necessity with military objective, dismissed the bridge’s profound cultural and historical value, and blatantly ignored the Hague Convention’s safeguards, exposing how international law can be manipulated as a convenient alibi for cultural obliteration under the veneer of legality ( De Vriese, 2018; ICTY, *Prosecutor v. Prlić et al.*, 2013). By elevating military judgment above legal and humanitarian obligations, this deferential approach flagrantly undermines international humanitarian law and human rights standards that demand strict accountability ( Drazewska, 2022). In his dissent, Judge Fausto Pocar strongly criticized the majority for adopting an alarmingly narrow interpretation, emphasizing that ‘imperative military necessity’ must be rigorously assessed in the context of cultural property. He stressed that clear proof is required to demonstrate that no feasible alternatives exist to achieve a comparable military advantage, a standard grounded in customary international law and enshrined in the 1999 Second Protocol ( ICTY, *Prosecutor v. Prlić et al.*, 2017b, Pocar, dissenting). Judge Pocar warned that reducing this standard to mere proportionality or the ambiguous concept of ‘military objectives’ systematically undermines protections for cultural heritage, enabling disproportionate attacks under a veneer of legality ( ICTY, *Prosecutor v. Prlić et al.*, 2017b, Pocar, dissenting). His dissent highlighted the pressing need for a contemporary standard capable of safeguarding cultural property against exploitation under the guise of operational convenience ( Drazewska, 2022).

**Reassessing the Protection of Cultural Heritage Under the ICC**

The ECCC in Case 002/01 further highlighted the indispensable role of proportionality within military necessity, stressing that forced transfers, even when justified on security or military grounds, must be strictly proportionate, represent the least intrusive means available, and remain directly linked to the legitimate objective pursued ( ICTY, *Prosecutor v. Prlić et al.*, 2017b, Pocar, dissenting). Moreover, the Chamber made clear that those who engineered the circumstances necessitating such measures cannot invoke necessity, and evidence of advance planning or repeated conduct demonstrates that these measures were both unnecessary and grossly disproportionate, revealing how international law is too often twisted to justify the destruction of cultural heritage ( ICTY, *Prosecutor v. Prlić et al.*, 2017b, Pocar, dissenting).

With respect to the military necessity exception, the relevant provisions adopt the narrower “military objectives” exclusion rather than the broader formulation of Article 52 of Additional Protocol I, which considers an object’s nature, location, purpose, or use, thereby aligning more closely with ICTY jurisprudence ( De Vriese, 2018). Nevertheless, this exclusion is far from perfect, as similar conduct may still be prosecuted under other crimes carrying broader exceptions, while the Rome Statute itself provides no precise definition of military necessity ( Frulli, 2011). This gap highlights the urgent need for the ICC to interpret Article 8(2)(b)(ix) in light of established international law and ICTY case law ( Brammertz *et al.*, 2016). The framework creates a clear asymmetry between attacker and defender: attackers may invoke military necessity only when cultural heritage qualifies as a military objective, whereas defenders face no sanction for transforming cultural property into a military objective ( De Vriese, 2018). The inconsistency becomes stark when compared with Article 8(2)(b)(xxiii), which prohibits the use of human shields but lacks a parallel prohibition against the use of cultural shields, a loophole defenders have exploited, as was witnessed during the Gulf War. By contrast, the ICTY *in Prlić* rejected the notion that mere “use,” absent consideration of location, suffices to render cultural property a military objective. Although the ICTY Statute contained no provision equivalent to Article 8(2)(b)(iv) of the Rome Statute, prosecutions were still pursuable under the Statute’s general provisions of Article 3. Article 8(2)(b)(xiii) of the Rome Statute criminalizes the destruction or seizure of enemy property unless “imperatively demanded by the necessities of war,” a standard that requires a proof that no feasible alternative exists to secure military safety, meaning broad or generic national security arguments cannot satisfy the threshold ( O’Keefe, 2010). This provision extends beyond grave breaches, as it does not require that destruction or seizure be extensive or wanton, and it applies whether the acts occur during combat, in belligerent states, or in occupied territory. Under the Elements of Crimes, the mental element is met when the perpetrator knew of the property’s protected status and of the existence of an armed conflict ( Rome Statute of the International Criminal Court, 1998). In sum, Article 8 of the Rome Statute affords explicit protection to cultural heritage, classifying its unlawful destruction as a war crime when it constitutes extensive destruction not justified by military necessity and carried out unlawfully and wantonly ( De Vriese, 2018). Nonetheless, the military necessity exception remains highly controversial, both conceptually and in practice, limiting the effectiveness of cultural heritage protection under international criminal law ( Rome Statute of the International Criminal Court, 1998). The notion of a military objective under Article 8(2)(b)(v) of the Rome Statute, and implicitly within Article 3(c) of the ICTY Statute, remains complex and insufficiently defined. As Abtahi notes, while Article 18 of the Fourth Geneva Convention invokes the term without clarification, Article 8(1) of the 1954 Hague Convention provides only a partial account through illustrative examples ( Abtahi, 2015). The protection of cultural heritage as civilian property thus proves inherently limited, since civilian objects may be reclassified as military objectives by virtue of their nature, location, purpose, or use—particularly when their destruction confers a military advantage, as articulated in Article 52(2) of Additional Protocol I. Consequently, cultural heritage can be deemed a military objective for reasons that extend beyond its direct military use ( De Vriese, 2018).

It is undeniable that the jurisprudence has long struggled with the ambiguous scope of military necessity in relation to cultural property. Tribunals have repeatedly rejected claims that monuments were employed militarily, signaling that assessing crimes against cultural heritage cannot be reduced merely to the notion of “military objectives.” Instead, principles such as proportionality, neutrality, and the protection of undefended places must guide accountability. Customary international law limits lawful attacks on cultural property to instances where such property constitutes a genuine military objective, reflected in Articles 8(2)(b)(ix) and 8(2)(e)(iv) of the Rome Statute and echoed in instruments such as UNTAET Regulation 2000/15 and the Statute of the Iraqi Special Tribunal (2003). It is particularly telling that the U.S. Military Commissions Act aligns with this standard, restricting jurisdiction over attacks on religious, educational, artistic, scientific, and historic buildings to cases where they are actively used militarily or otherwise qualify as military objectives. Article 52(2) of Additional Protocol I reinforces this framework, defining a military objective as an object that makes an effective contribution to military action and whose destruction offers a definite advantage, modernizing the classical “imperative necessity” rule ( O’Keefe, 2010).

The ICTY has directly confronted these challenges. In Strugar and Hadžihasanović (ICTY, *Prosecutor v. Hadžihasanović & Kubura*, 2006), it established that attacks on cultural property are permissible only when the property is contemporaneously used for military purposes at the time of attack. The Appeals Chambers have refined this stance: the Strugar appeal emphasized that the attack on Dubrovnik’s Old Town was “not justified by any military necessity,” exposing the inadequacy of a simplistic military-use test. The Brđanin case confirmed that prosecutions must establish that destruction was not justified by military necessity, with Article 52 of Additional Protocol I providing the interpretive guidance. However, practically, defenders of cultural heritage remain vulnerable to legal loopholes. ( ICTY, *Prosecutor v. Hadžihasanović & Kubura*, 2006; ICTY, *Prosecutor v. Strugar*, 2003c; ICTY, *Prosecutor v. Brđanin*, 2007).

The principle of military necessity evidently holds limited efficacy in protecting cultural property. The case of Prosecutor v. Al Mahdi illustrates this, since the ICC categorically rejected any military rationale, recognizing the deliberate destruction of Timbuktu’s mausoleums as inherently unlawful; the defendant’s admission of guilt underscored the primacy of cultural heritage as an independent value. ICTY rulings in Kordić and Čerkez and Jokić further demonstrate that attacks on cultural sites are typically disproportionate or unjustified, rendering military necessity arguments largely irrelevant ( O’Keefe, 2010). It is increasingly clear that military necessity operates less as a legitimate legal doctrine than as a convenient smokescreen, rarely persuasive and morally untenable when invoked to justify the deliberate targeting of cultural heritage.

The principle of military necessity, enshrined in the 1954 Hague Convention and its 1999 Second Protocol, was designed to allow exceptional flexibility while safeguarding cultural heritage. Yet, from Iraq’s invasion of Kuwait to the Balkan conflicts, it has often been misused, creating a legal loophole that justifies destruction under the guise of military imperatives. Such exploitation underscores the structural fragility of international law when humanistic and cultural obligations are subordinated to transient military advantage ( Forrest, 2007).

International jurisprudence has emphasized the limited scope of military necessity. The ICTY, in cases such as Blaškić, Strugar, and Hadžihasanović, stressed that cultural property loses protection only if actively used for military purposes, and attacks must be proportional. Similarly, the ICC in *Prosecutor v. Al Mahdi* affirmed that heritage possesses intrinsic value independent of military considerations. These rulings underscore that military necessity cannot serve as a blanket justification ( Cunliffe, Muhesen, & Lostal, 2016).

Despite the 1999 Second Protocol narrowing its application to cases where no feasible alternative exists, ambiguity remains regarding “military objectives” and proportionality ( Forrest, 2007). A further concern is the asymmetry between attackers and defenders. While attacking forces are constrained by the requirement that cultural heritage qualifies as a military objective, defenders may exploit such sites for military purposes without facing sanctions. This imbalance has real-world consequences, as evidenced in conflicts like the Gulf War, where cultural property was used strategically without legal recourse for those harmed by the attacks. Although ICTY rulings, such as in Prlić, rejected the idea that mere “use” alone converts heritage into a military objective, these protections remain inconsistently applied, leaving room for exploitation.

Accountability mechanisms are limited, allowing states and armed groups to invoke military necessity with minimal consequences. Scholars like Merryman have criticized the clause as outdated, incompatible with modern humanitarian law. Advanced military technology could enable greater protection, yet necessity continues to be invoked to justify attacks, reflecting a gap between legal norms and operational conduct ( McGeorge, 2016; Merryman, 1986; Cunliffe, Muhesen, & Lostal, 2016; O’Keefe, 2010).

Modern military technology could enhance protection, yet the pretext of necessity continues to justify attacks, revealing a persistent gap between military objectives and protected cultural sites. Indeed, military necessity continues to be invoked to justify attacks. As Eisenhower warned, the term is too often used to disguise convenience, negligence, or deliberate indifference. This ongoing practice highlights a critical misalignment between normative law and operational conduct, where the pretext of necessity undermines the protection of humanity’s shared cultural heritage ( Cunliffe, Muhesen, & Lostal, 2016; McGeorge, 2016; O’Keefe, 2010).

In conclusion, the role of military necessity in international law remains highly contentious. Intended as a narrow exception, it has too often functioned as a permissive doctrine enabling the destruction of cultural property, leaving profound gaps in accountability. Closing these loopholes and strengthening accountability is both a legal and moral imperative, recognizing cultural property as an indispensable element of human civilization rather than a disposable tool of military gain.

## Conclusion

The doctrine of military necessity in international law remains deeply problematic. While conceived as a narrow exception, it has too often served as a permissive justification for the destruction of cultural property, undermining accountability and exposing systemic weaknesses in both treaty law and judicial practice. The 1954 Hague Convention and its 1999 Second Protocol provide foundational protections, yet vague provisions—such as the “adequate distance” requirement and the conditional allowance for attacks when “no feasible alternative” exists—leave room for subjective interpretation and opportunistic application. International jurisprudence has been inconsistent: while cases like Strugar and Al Mahdi illustrate a more restrictive and principled approach, decisions such as Prlić highlight the continued vulnerability of heritage protection to uneven enforcement.

To remedy these deficiencies, the scope of military necessity should be further restricted through a formal amendment to Article 6 of the Second Protocol, making it explicit that such exceptions apply only when no reasonable alternative exists and requiring independent verification by impartial officers. Ambiguous terms like “adequate distance” and “feasible alternative” should be precisely defined, possibly through detailed operational guidelines or an interpretive annex. Moreover, a new protocol could establish robust enforcement mechanisms, including mandatory reporting of attacks affecting cultural heritage, international oversight, and clear liability for commanders or states that unjustifiably invoke military necessity. Proportionality assessments should be standardized, requiring documented evaluations of anticipated military advantage versus potential harm to cultural property and civilians, subject to judicial review to ensure compliance. Finally, the establishment of an expert advisory body could guide tribunals in the consistent interpretation of military necessity, preventing divergent applications across international cases.

Filling these gaps is both a legal and ethical necessity. By imposing stricter limits on military necessity, enhancing proportionality standards, and strengthening accountability, cultural heritage can be effectively safeguarded. Without such reforms, the protection of cultural property risks remaining dependent on operational convenience, perpetuating a pattern in which military priorities continue to overshadow humanitarian obligations.

### Ethical considerations

This study is a doctrinal legal study; therefore, it was not conducted on human or animal participants. The research did not require ethical approval. The research is consistent with the ethical conduct of scientists and ensures academic integrity according to the standards of legal science.

## Data Availability

All the data underlying the results of this study were obtained from publicly accessible legal sources, including international treaties, conventions, protocols, and international tribunal judicial decisions. No new data sets were created or analyzed in this study. The article references all sources. This article is doctrinal legal research and does not include or involve clinical trials, animal studies, or observational/qualitative research; therefore, reporting guidelines from the EQUATOR Network (CONSORT, ARRIVE, STROBE, COREQ, or SRQR) cannot be applied. The research is consistent with accepted principles of academic and legal research.
