# Supplementary material for: Between Protection and Destruction: The Legal Tension Between Military Necessity and Cultural Heritage Protection in International Humanitarian Law
Source: F1000Res. 2025 Oct 17;14:1032. Originally published 2025 Oct 3. [Version 2] doi: 10.12688/f1000research.170650.2 (PMC12529059; doi:10.12688/f1000research.170650.2)
Supplement: Peer review history

# Reviewer report: Reviewer response for version 2

**DOI:** 10.5256/f1000research.189770.r431199

**Published:** 2025-11-21

**Authors:** Farah Julie Yassine

**Affiliations:** 1 Kuwait International Law School, Doha, Kuwait

The article is well-written, clearly presented, and supported by current and relevant literature. The study structure is consistent, and the methodology is convenient, providing adequate details for replication. The analysis is clear and based on well-known legal sources. The article meets the requirements for reproducibility because it does not need any underlying source data for this doctrinal legal study. The arguments and evidence are well provided to support the conclusions, and the limitations are adequately explained. In general, the papers meets the standards of scientific soundness that are expected for indexing.

Is the work clearly and accurately presented and does it cite the current literature?

Yes

If applicable, is the statistical analysis and its interpretation appropriate?

Not applicable

Are all the source data underlying the results available to ensure full reproducibility?

No source data required

Is the study design appropriate and is the work technically sound?

Yes

Are the conclusions drawn adequately supported by the results?

Yes

Are sufficient details of methods and analysis provided to allow replication by others?

Yes

Reviewer Expertise:

NA

I confirm that I have read this submission and believe that I have an appropriate level of expertise to confirm that it is of an acceptable scientific standard.

# Reviewer report: Reviewer response for version 2

**DOI:** 10.5256/f1000research.189770.r425050

**Published:** 2025-10-22

**Authors:** Ali Almatrooshi

**Affiliations:** 1 Dubai Police Academy, Dubai, United Arab Emirates

no comments. The manuscript is okay to be indexed.

Is the work clearly and accurately presented and does it cite the current literature?

Yes

If applicable, is the statistical analysis and its interpretation appropriate?

Yes

Are all the source data underlying the results available to ensure full reproducibility?

Yes

Is the study design appropriate and is the work technically sound?

Yes

Are the conclusions drawn adequately supported by the results?

Yes

Are sufficient details of methods and analysis provided to allow replication by others?

Yes

Reviewer Expertise:

International Public Law

I confirm that I have read this submission and believe that I have an appropriate level of expertise to confirm that it is of an acceptable scientific standard.

# Reviewer report: Reviewer response for version 2

**DOI:** 10.5256/f1000research.189770.r425049

**Published:** 2025-10-22

**Authors:** Jamal Barafi

**Affiliations:** 1 University of Sharjah, Sharjah, United Arab Emirates

I have reviewed the revised manuscript and the authors’ responses. I approve the revised version and have no further comments.

Is the work clearly and accurately presented and does it cite the current literature?

Yes

If applicable, is the statistical analysis and its interpretation appropriate?

Not applicable

Are all the source data underlying the results available to ensure full reproducibility?

Yes

Is the study design appropriate and is the work technically sound?

Yes

Are the conclusions drawn adequately supported by the results?

Yes

Are sufficient details of methods and analysis provided to allow replication by others?

Yes

Reviewer Expertise:

Public International Law, International Human Rights Law, international humanitarian law, and international organization law.

I confirm that I have read this submission and believe that I have an appropriate level of expertise to confirm that it is of an acceptable scientific standard.

# Reviewer report: Reviewer response for version 1

**DOI:** 10.5256/f1000research.188138.r421057

**Published:** 2025-10-15

**Authors:** Ali Almatrooshi

**Affiliations:** 1 Dubai Police Academy, Dubai, United Arab Emirates

Public Review Summary (for indexing on article page)

 This is a well-researched and compelling article that makes an important scholarly contribution to the understanding of how military necessity interacts with the protection of cultural heritage under international humanitarian law. The study is comprehensive, methodologically sound, and exceptionally well written. It successfully integrates treaty analysis, international jurisprudence, and scholarly perspectives to present a balanced and insightful argument. I find the article ready for indexing in its current form and recommend acceptance with no revisions.

 1. Summary of the Article

 The article titled “Between Protection and Destruction: The Legal Tension Between Military Necessity and Cultural Heritage Protection in International Humanitarian Law” provides a profound and systematic analysis of the complex relationship between the principles of military necessity and cultural heritage protection. The authors employ a doctrinal and analytical legal method, examining key international treaties including the 1954 Hague Convention, the 1999 Second Protocol, and the Rome Statute of the International Criminal Court as well as leading jurisprudence from the ICTY and ICC, such as Blaškić, Strugar, Hadžihasanović, Prlić, and Al Mahdi.

 The study finds that, although legal frameworks strive to balance military objectives with humanitarian obligations, the concept of “imperative military necessity” often creates loopholes that allow the destruction of cultural property. The authors conclude that these structural weaknesses in international law must be addressed to ensure that cultural heritage remains protected even during armed conflict.

 2. Overall Assessment

 This article demonstrates a high level of academic rigor and originality. The argument is logically developed, deeply informed by both primary legal materials and secondary scholarship, and expressed with clarity and precision. The authors’ engagement with international case law, particularly ICTY and ICC judgments, is exemplary, reflecting a sophisticated understanding of how doctrine translates into practice. The writing is articulate and scholarly, with clear structure and consistent referencing. The study contributes meaningfully to the academic discourse on international humanitarian law, cultural heritage, and military operations, and its conclusions are well-founded and persuasive. The article also excels in synthesizing historical evolution with contemporary application—moving seamlessly from the Lieber Code (1863) and Hague Conventions (1907) to modern accountability mechanisms under the Rome Statute. It provides a balanced critique, highlighting both the strengths and limitations of existing frameworks.

 3. Strengths of the Article

 • Comprehensive coverage of doctrinal sources and jurisprudence.

 • Excellent analytical depth, particularly in linking military necessity and proportionality under treaty law.

 • Clear structure and coherence, with each section logically building on the previous.

 • Strong use of primary sources, including treaties and international tribunal decisions.

 • Engagement with contemporary scholarship, situating the discussion in modern debates about heritage protection and armed conflict.

 • High academic quality, meeting the publication standards of F1000Research.

 4. Recommendation

***Decision:*** ***Accept — No Changes Required***

 The article is a model of doctrinal legal research and a valuable contribution to international legal scholarship. It demonstrates academic excellence, conceptual clarity, and legal precision. I find the analysis comprehensive and persuasive, and I see no need for revision.

 I therefore recommend this article for indexing in its current form.

Is the work clearly and accurately presented and does it cite the current literature?

Yes

If applicable, is the statistical analysis and its interpretation appropriate?

Yes

Are all the source data underlying the results available to ensure full reproducibility?

Yes

Is the study design appropriate and is the work technically sound?

Yes

Are the conclusions drawn adequately supported by the results?

Yes

Are sufficient details of methods and analysis provided to allow replication by others?

Yes

Reviewer Expertise:

International Public Law

I confirm that I have read this submission and believe that I have an appropriate level of expertise to confirm that it is of an acceptable scientific standard.

# Reviewer report: Reviewer response for version 1

**DOI:** 10.5256/f1000research.188138.r421050

**Published:** 2025-10-13

**Authors:** Jamal Barafi

**Affiliations:** 1 University of Sharjah, Sharjah, United Arab Emirates

This article critically analyzes the conflict between the principle of military necessity and the safeguarding of cultural heritage within the framework of international humanitarian law (IHL). It follows the history of military necessity from the 1863 Lieber Code to the 1907 Hague Conventions, where it was officially recognized, and then to the 1954 Hague Convention and the 1999 Second Protocol, where it was added to the list of rules. The authors argue that while military necessity was intended as a narrowly defined exception, it has often been misused to justify the destruction of cultural property during armed conflict.

 The topic is interesting and relevant. The article was written in clear and understandable language, and a significant number of international court cases related to the principle of necessity were addressed.

**However, some points must be noted:**

It is recommended to improve some expressions, such as:  1 - **'have endeavored **to restrict these justifications...' to: 'international tribunals, such as the International Criminal Tribunal for the Former Yugoslavia (ICTY) and the International Criminal Court (ICC), have sought to limit...'

 2 **-Clarification that the withdrawal intended here is the withdrawal of immunity: **reasonable advance notice should be provided when possible, and the withdrawal must last only as long as necessary....

 3- Modify the sentence and delete the repetition on page 6: neutralization offers a definite military advantage advantage. 

 4 - In several sections of the manuscript, the idea that military necessity is used as a pretext for the destruction of cultural heritage is repeated without introducing new legal insights or illustrative examples. I recommend reducing this repetition or enriching the discussion with additional legal analysis.

 5 - To make the text more fluid in some sections, it is recommended to break up large paragraphs that exceed 15 lines. This will make the text easier to read and help readers follow the legal arguments more clearly.

**Regarding the conclusion,** more concrete recommendations should be included — for instance, proposing amendments to specific legal provisions or introducing new protocols to address existing legal loopholes, with a clear delineation of challenges and limits of these suggested reforms.

Is the work clearly and accurately presented and does it cite the current literature?

Yes

If applicable, is the statistical analysis and its interpretation appropriate?

Not applicable

Are all the source data underlying the results available to ensure full reproducibility?

Yes

Is the study design appropriate and is the work technically sound?

Yes

Are the conclusions drawn adequately supported by the results?

Yes

Are sufficient details of methods and analysis provided to allow replication by others?

Yes

Reviewer Expertise:

International law, international human rights law, international humanitarian law, international organization law.

I confirm that I have read this submission and believe that I have an appropriate level of expertise to confirm that it is of an acceptable scientific standard.
